# Beneficial Effect of Herbal Formulation KM1608 on Inflammatory Bowl Diseases: A Preliminary Experimental Study

**DOI:** 10.3390/molecules23082068

**Published:** 2018-08-17

**Authors:** Myoung-Sook Shin, Sang-Back Kim, Jaemin Lee, Han-Seok Choi, Jimin Park, Jun Yeon Park, Sullim Lee, Gwi Seo Hwang, Bon Am Koo, Ki Sung Kang

**Affiliations:** 1 College of Korean Medicine, Gachon University, Seongnam-si, Gyeonggi-do 13120, Korea; ms.shin@gachon.ac.kr (M.-S.S.); jaemin.lee426@gmail.com (J.L.); seoul@gachon.ac.kr (G.S.H.); 2 New Drug Research Team, Kolmar, Korea Co. Ltd, Sandan-gil, Jeonui-myeon, Sejong-si 30003, Korea; m302@kolmar.co.kr (S.-B.K.); fcosmos@kolmar.co.kr (H.-S.C.); jimpark@kolmar.co.kr (J.P.); 3 Department of Food Science and Biotechnology, Kyonggi University, Suwon-si, Gyeonggi-do 16227, Korea; rhemf@hanmail.net; 4 Department of Life Science, College of Bio-Nano Technology, Gachon University, Seongnam-si, Gyeonggi-do 13120, Korea; sullimlee@gachon.ac.kr

**Keywords:** anti-inflammatory, *Aucklandia lappa* DC., ulcerative colitis, *Terminalia chebula* Retz., *Zingiber officinale* Roscoe

## Abstract

*Aucklandia lappa* DC., *Terminalia chebula* Retz and *Zingiber officinale* Roscoe have been traditionally used in east Asia to treat chronic diarrhea and abdominal pain. This study aimed to evaluated the anti-inflammatory activity of KM1608, which is composed of three natural herbs in a mouse model of dextran sodium sulfate (DSS)-induced ulcerative colitis. The anti-inflammatory activity and underlying mechanism were assessed in vitro using LPS-treated RAW264.7 cells. The in vivo effect of KM1608 on DSS-induced colitis was examined after oral administration in mice. KM1608 significantly inhibited the inflammatory mediators such as nitric oxide, interleukin (IL)-6, monocyte chemotactic protein 1 (MCP-1) and tumor necrosis factor (TNF)-α in LPS-treated RAW264.7 cells. The inhibitory effect of KM1608 was attributed to the reduction of Akt phosphorylation in the LPS-treated cells. In the mouse model, oral administration of KM1608 significantly improved DSS-induced colitis symptoms, such as disease activity index (DAI), colon length, and colon weight, as well as suppressed the expression of IL-6, TNF-α, and myeloperoxidase (MPO) in the DSS-induced colitis tissues. Taken together, KM1608 improved colitis through the regulation of inflammatory responses, suggesting that KM1608 has potential therapeutic use in the treatment of inflammatory diseases.

## 1. Introduction

Inflammation, one of the biological defense processes against external stimuli, is a complex physiological response to tissue damage. When inflammatory reactions occur, inflammatory mediators, such as nitric oxide (NO), tumor necrosis factor (TNF)-α, IL-6, prostaglandin E2 (PGE2), and monocyte chemotactic protein 1 (MCP-1) are secreted [1,2,3,4]. However, persistent inflammatory responses are the cause of chronic inflammatory diseases, such as allergies, atherosclerosis, autoimmune diseases, and tumors [2].

Inflammatory bowel disease (IBD) is a set of chronic inflammatory conditions that occur in the intestine, which includes Crohn’s disease and ulcerative colitis. Genetic and environmental factors are known to play a complex role in IBD pathogenesis, although the mechanism remains unclear. In general, the main symptoms in patients with IBD include bleeding, diarrhea, urgency, and abdominal pain [5,6]. Currently, medications for IBD include 5-aminosalicylic acid (5-ASA); a steroid, such as a prednisolone; immunomodulatory drugs, such as cyclosporine and tacrolimus; and neutralizing antibodies against TNF-α. However, 20–40% of patients treated with these drugs will undergo colonic resection owing to drug failure or side effects [7,8]. Thus, there have been many attempts to develop more-effective drugs with minimum side effects to treat colitis.

Over the past decade, herbal extracts and their active ingredients have been studied in the development of anti-inflammatory agents [9,10,11,12,13,14,15]. *Aucklandia lappa* DC., *Terminalia chebula* Retz and *Zingiber officinale* Roscoe are well-known medicinal plants widely used in the traditional medicine of east Asia. The major components of these three natural products are reported as follows; *T. chebula*: gallic acid, corilagin, ellagic acid, 1,2,3,4,6-pentagalloyl-glucose [16], *A. lappa*: dehydrocostus lactone [17], and *Z. officinale*: 6-gingerol [18]. Many studies have reported that the extracts and active ingredients of *A. lappa*, *T. chebula* and *Z. officinale*, such as dehydrocostus lactone (DCL), ellagic acid (EA), and 6-gingerol (6G) respectively, suppress iNOS gene/protein expression, NO production, PGE2 production, and TNF-α production in lipopolysaccharide (LPS)-stimulated RAW264.7 cells [19,20,21] and in a rat model of carrageenan-induced paw edema [22].

Most earlier studies have focused on the individual extracts and active ingredients of natural products. However, through an in vitro screening of anti-inflammatory plants, we recently composed a potent herbal formulation (KM1608) containing *A. lappa*, *T. chebula* and *Z. officinale* to treat IBD. Therefore, the present study aimed to examine the chemical properties of KM1608 and assess its anti-inflammatory effect in vitro and DSS-induced ulcerative colitis in vivo.

## 2. Results and Discussion

### 2.1. HPLC Profile of KM1608

**Figure 1:**
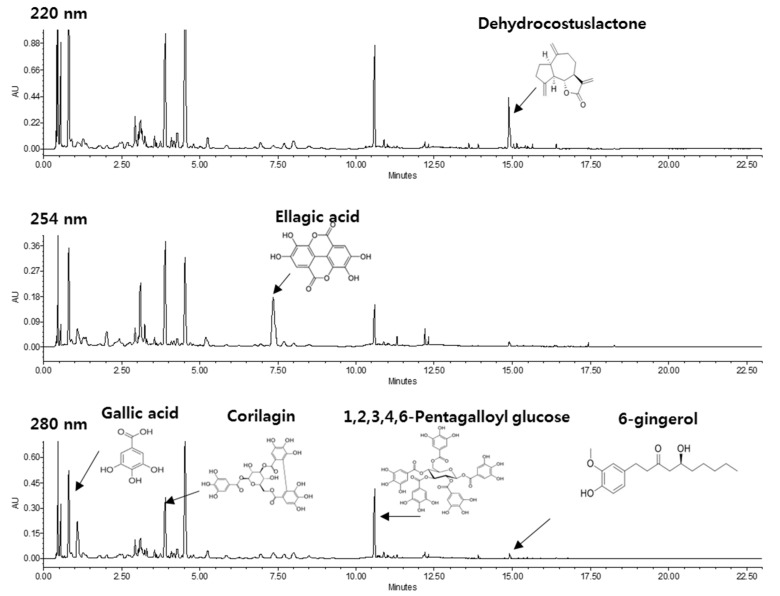
HPLC profiles of KM1608. The peak of dehydrocostus lactone at 220 nm, ellagic acid at 254 nm, and gallic acid, corilagin, 1,2,3,4,6-pentagalloyl glucose, and 6-gingerol at 280 nm in KM1608 were compared with those of their respective standard compounds.

Through HPLC analysis we showed that KM1608 contained a wide variety of phytochemicals (Figure 1). Among them, DCL, EA, and 6G, contained in *A. lappa*, *T. chebula* and *Z. officinale*, respectively, are active compounds possessing anti-inflammatory activity. KM1608 contained 0.68–1.2%, 0.94–1.5%, and 0.1–0.2% of DCL, EA, and 6G, respectively (Table 1). Also, gallic acid, corliagin, 1,2,3,4,6-pentagalloyl-glucose were detected from HPLC analysis and these three compounds have been proven to have an inhibitory effect on DSS-induced ulcerative colitis or other biological activity [23,24,25]. From these, we predicted that KM1608 might exhibit anti-inflammatory activity.

### 2.2. Anti-Inflammatory Activity of KM1608 in LPS-Induced RAW264.7 Cells

**Figure 2:**
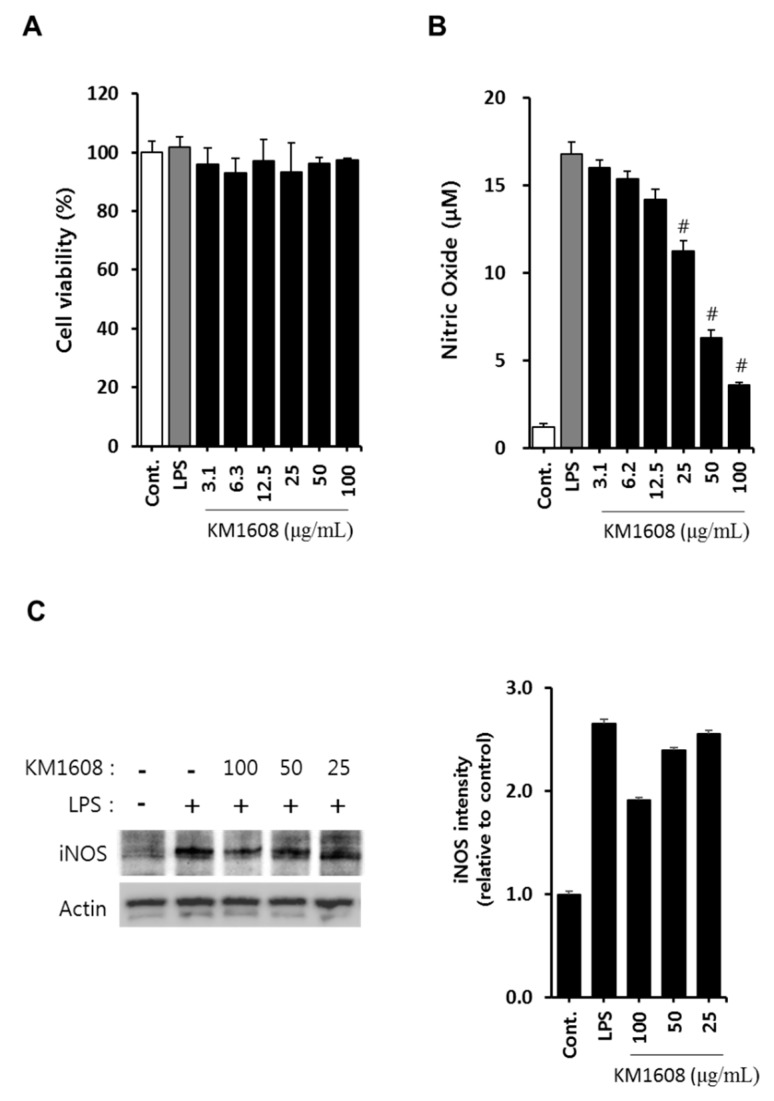
Effect of KM1608 on nitric oxide production and iNOS protein expression in lipopolysaccharide (LPS)-induced RAW264.7 cells. RAW264.7 cells (1 × 10^5^ cells/well, 96-well plate) were treated with various concentrations of KM1608 for 2 h and subsequently with LPS (250 ng/mL) for 18 h. The cytotoxicity was determined using the CCK-8-based colorimetric assay (**A**). After incubation, cell supernatants were collected and assayed for nitric oxide using the Griess reagent (**B**). RAW264.7 cells (2 × 10^6^ cells/well, 6-well plate) were treated with various concentrations of KM1608 for 2 h and subsequently with LPS (250 ng/mL) for 18 h (**C**). Control group treated with DMSO at 0.1%. iNOS protein level was measured by immunoblotting with the specific antibodies. The bar chart displays the intensity of immunoblot bands visualized using the Image J software. Data are presented as the mean ± SD of three independent experiments. ^#^ *p* < 0.05 vs. the LPS group.

To investigate the anti-inflammatory activity of KM1608, we first examined its cytotoxicity on RAW264.7 cells. RAW264.7 cells were treated with various concentrations of KM1608 for 2 h and further treated with LPS (250 ng/mL) for 18 h. As shown in Figure 2A, the treatment with KM1608 at 3.1–100 μg/mL did not exert cytotoxicity in RAW264.7 cells.

**Figure 3:**
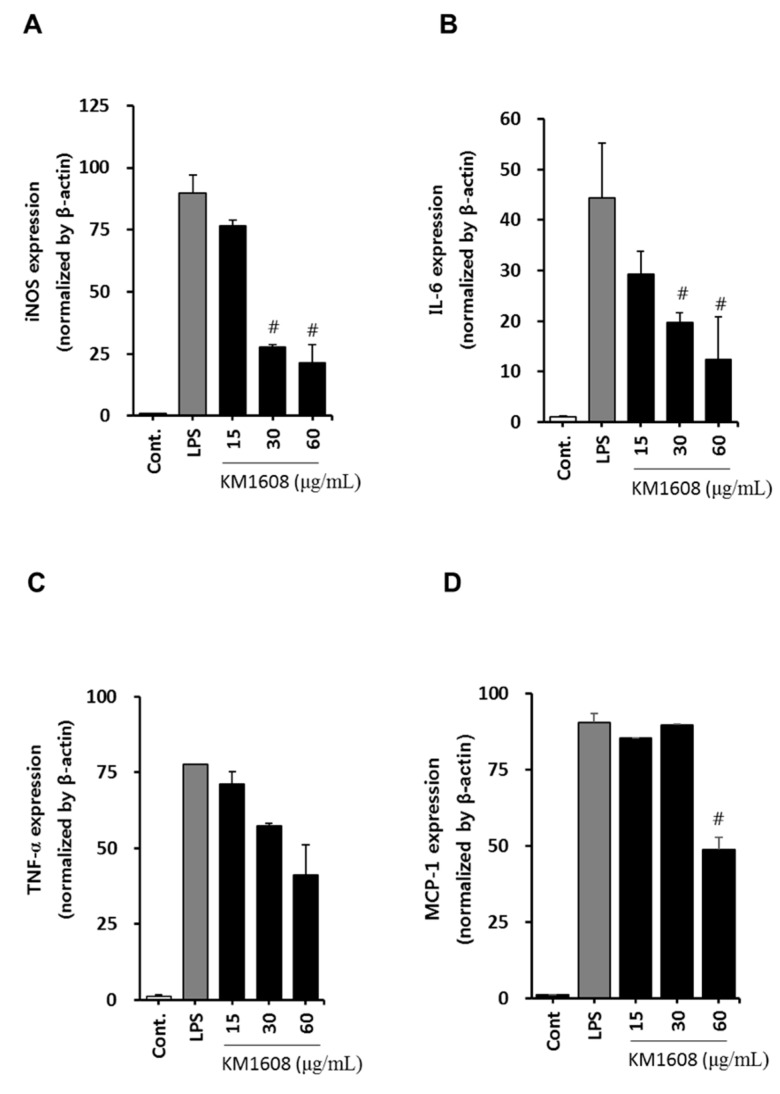
Effect of KM1608 on iNOS, IL-6, TNF-α and MCP-1 mRNA expression in LPS-stimulated RAW264.7 cells. RAW264.7 cells (2 × 10^6^ cells/well, 6-well plate) were treated with various concentrations of KM1608 for 2 h and subsequently with LPS (250 ng/mL) for 4 h. IL-6 and TNF-α mRNA levels were measured using qRT-PCR (**A**–**D**). Control group was treated with DMSO at 0.1%. Data are presented as the mean ± SD of three independent experiments. ^#^ *p* < 0.05 vs. the LPS group.

**Figure 4:**
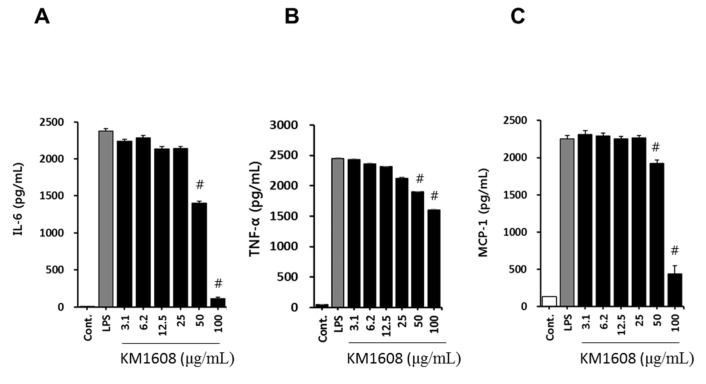
Effect of KM1608 on IL-6, TNF-α and MCP-1 production in LPS-stimulated RAW264.7 cells. RAW264.7 cells (1 × 10^5^ cells/well, 96-well plate) were treated with various concentrations of KM1608 for 2 h and subsequently with LPS (250 ng/mL) for 18 h. IL-6, TNF-α and MCP-1 concentrations were measured by using ELISA (**A**–**C**). Control group was treated with DMSO at 0.1%. Data are presented as the mean ± SD of three independent experiments. ^#^ *p* < 0.05 vs. the LPS group.

The macrophage, which differentiates from blood mononuclear cells, releases inflammation mediators, such as nitric oxide (NO), PGE2, TNF-α, IL-1, and IL-6. NO is produced from l-arginine by the nitric oxide synthases (NOS), neuronal NOS, endothelial NOS, and inducible NOS (iNOS) present in various tissues and cells. Because iNOS gene expression in the macrophage is induced by bacterial endotoxin or inflammatory stimuli, we first investigated whether KM1608 inhibits NO production in LPS-induced RAW264.7 cells. As shown in Figure 2B,C, treatment with KM1608 at 25–100 μg/mL significantly suppressed LPS-induced nitric oxide production and iNOS protein expression. The levels of other pro-inflammatory mediators, namely IL-6, TNF-α, and MCP-1, were also analyzed in LPS-induced RAW264.7 cells. As shown in Figure 3, KM1608 inhibited LPS-induced iNOS, IL-6, TNF-α and MCP-1 gene expression in a concentration-dependent manner. KM1608 at 30–60 μg/mL significantly decreased LPS-induced iNOS, IL-6 and TNF-α gene expression levels (Figure 3A,C), and KM1608 at 60 μg/mL decreased MCP-1 gene expression (Figure 3D). Similarly, IL-6, TNF-α and MCP-1 production after LPS stimulation were significantly reduced by KM1608 treatment at at 50–100 μg/mL (Figure 4A–C). Taken together, these results indicated that KM1608 could possess anti-inflammatory activity in LPS-induced RAW264.7 cells.

### 2.3. Effect of KM1608 on Akt Phosphorylation in LPS-Induced RAW264.7 Cells

**Figure 5:**
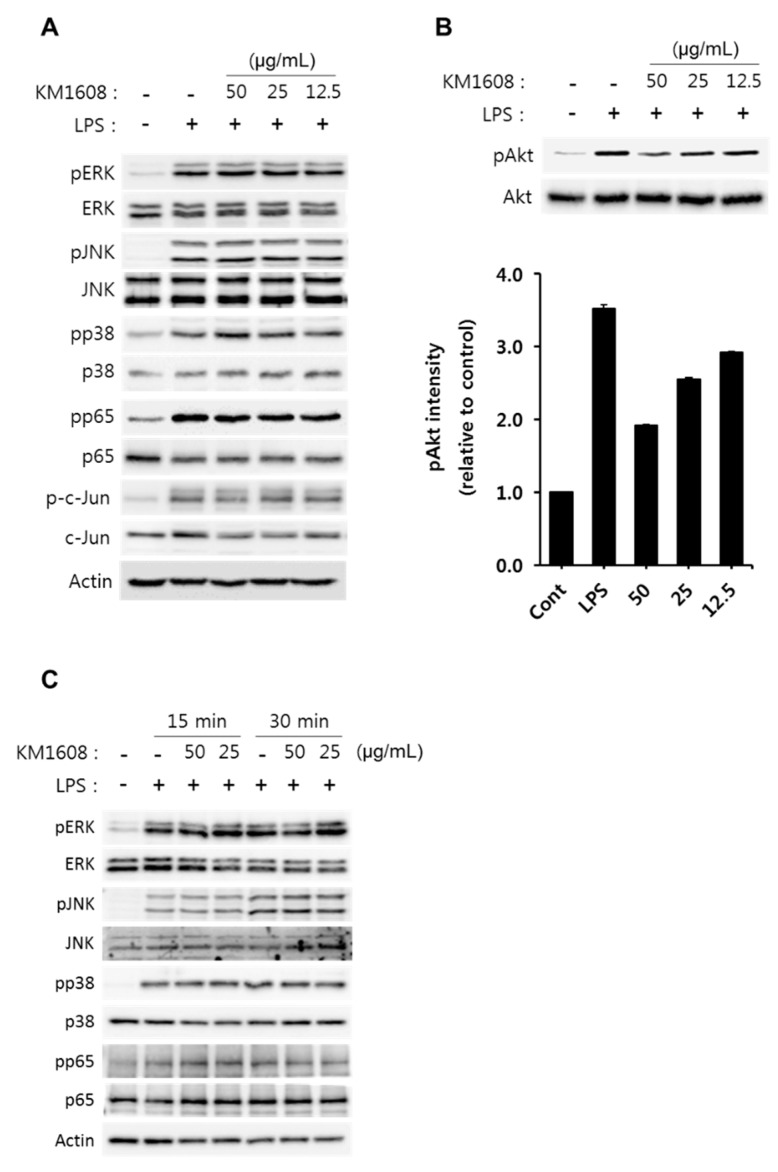
Effect of KM1608 on the phosphorylation of MAPKs and Akt in RAW264.7 cells. RAW264.7 cells (2 × 10^6^ cells/well, 6-well plate) were treated with various concentrations of KM1608 for 2 h and subsequently with LPS (250 ng/mL) for 1 h (**A**,**B**). RAW264.7 cells (2 × 10^6^ cells/well, 6-well plate) were treated with various concentration of KM1608 for 2 h and subsequently with LPS (250 ng/mL) for 15 min or 30 min (**C**). Control group was treated with DMSO at 0.05%. Whole cell-lysates were immunoblotted with the indicated specific-antibodies. β-Actin served as an internal loading control. Whole Western blot membranes of Figure 5C; See Figure S1. The bar chart displays the intensity of immunoblot bands visualized using the Image J software. Data are presented as the mean ± SD of three independent experiments.

To elucidate the underlying anti-inflammatory mechanisms of KM1608, we investigated several intracellular signaling pathways in LPS-induced RAW264.7 cells. As shown in Figure 5A, LPS strongly induced the phosphorylation of MAPKs (ERK1/2, JNK1/2, p38), NF-κB p65, and c-Jun for 1 h. However, pretreatment of KM1608 for 2 h did not affect LPS-induced ERK, JNK and p38 phosphorylation. Similarly, p65 and c-Jun phosphorylation were also not altered by KM1608 in the LPS-stimulated RAW264.7 cells. It is well-known that PI3K/Akt signaling is involved in the regulation of pro-inflammatory genes and that Akt is a downstream molecule of PI3K in macrophages [26,27]. Following the treatment of cells with LPS for 1 h, Akt was strongly phosphorylated. However, LPS-induced Akt phosphorylation was diminished by pre-treatment with KM1608 in a concentration-dependent manner (Figure 5B). These findings suggested that KM1608 inhibited pro-inflammatory mediators (NO, IL-6, and MCP-1) by regulating the Akt signaling pathway. Also, we confirmed inhibitory effects of KM1608 on LPS-induced MAPKs and p65 phosphorylation in short term point. When the cells were stimulated by LPS for 15 min or 30 min, MAPKs and p65 phosphorylation increased by time. KM1608 treatment at 50 μg/mL or 25 μg/mL did not affect LPS signaling at 15 min, however, LPS-induced ERK phosphorylation was slightly decreased by KM1608 at 30 min (Figure 5C). This inhibited ERK signaling by KM1608 may involve a reduction of Akt phosphorylation, and more detailed studies should be continued in the future.

### 2.4. Effect of KM1608 on Clinical Signs of DSS-Induced Colitis in Mice

**Figure 6:**
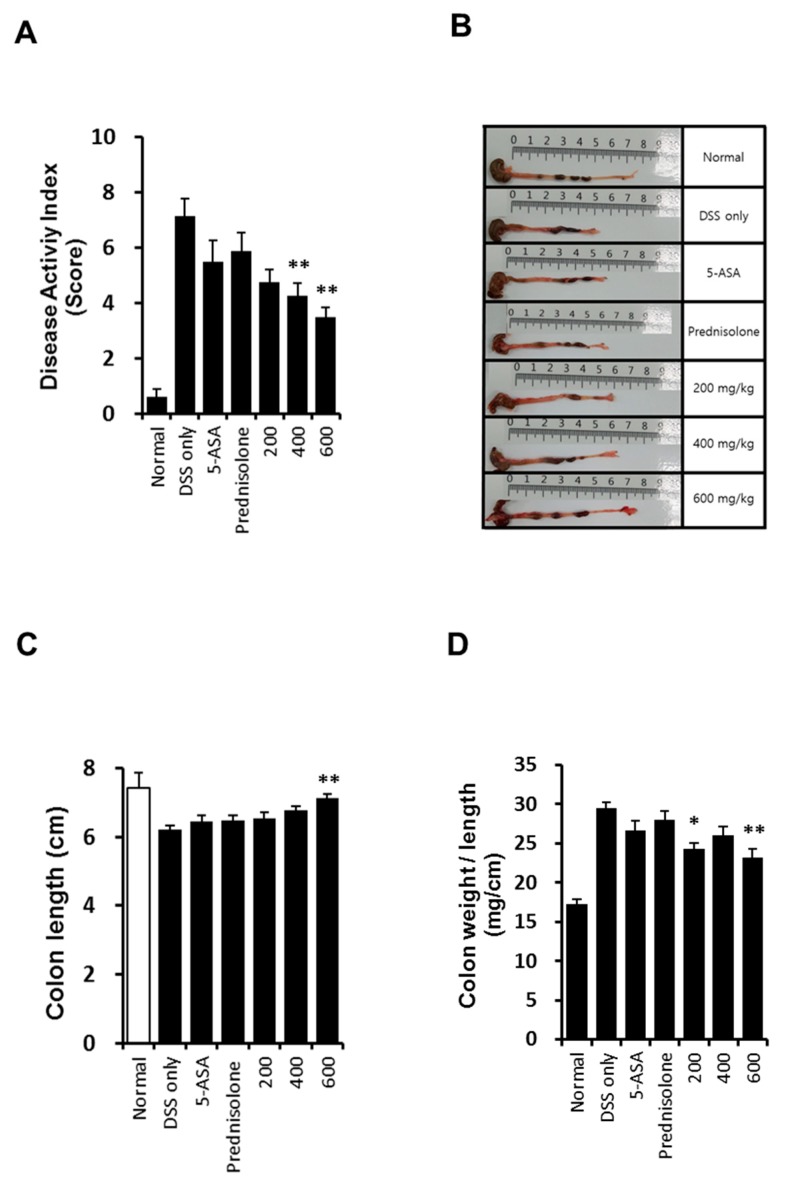
KM1608 improves the symptoms of DSS-induced colitis. Ulcerative colitis was induced in C57BL/6 mice by administering 1.7% DSS in the drinking water for 7 days. During this period, the mice also received KM1608 (200, 400, and 600 mg/kg), 5-ASA (200 mg/kg), or prednisolone (5 mg/kg) orally once a day. 5-ASA and prednisolone were used as reference drugs. DAI was scored in all seven of the study groups (**A**). Colons were harvested on day 7 and (**B**) colon length (**C**) and colon weight (**D**) were measured. * *p* < 0.05 or ** *p* < 0.01 vs. the DSS group.

**Figure 7:**
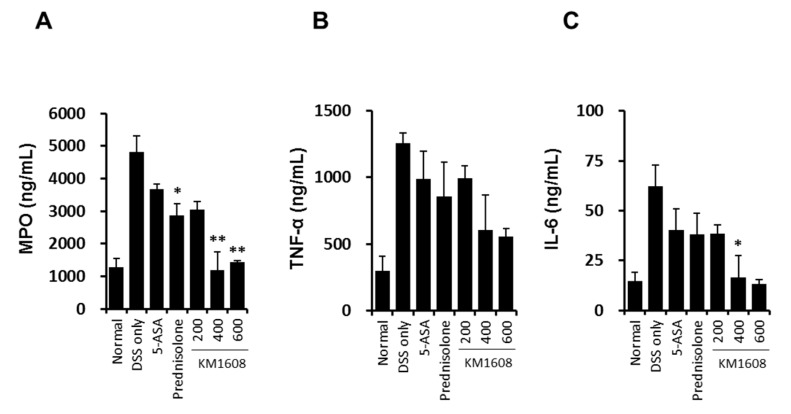
KM1608 inhibits the inflammatory factors involved in DSS-induced colitis. Ulcerative colitis was induced in C57BL/6 mice by administering 1.7% DSS in the drinking water for 7 days. During the period, the mice also received KM1608 (200, 400, and 600 mg/kg), 5-ASA (200 mg/kg), or prednisolone (5 mg/kg) orally once a day. MPO, TNF-α, and IL-6 levels in the colon tissue lysates were measured using ELISA kits (**A**–**C**). Data are presented as the mean ± SD of three independent experiments. * *p* < 0.05 or ** *p* < 0.01 vs. the DSS group.

To investigate the in vivo efficacy of KM1608 in IBD model, we established a mouse model of DSS-induced colitis. Clinical assessment of colitis was conducted using the DAI, which represents the sum of the highest score of each criterion (weight loss, stool consistency and rectal bleeding) during DSS treatment for 7 days (Figure 6A). KM1608 administration significantly ameliorated the severity of colitis in a dose-dependent manner (Figure 5A). DSS-treated mice showed a significant shortening of colon length (Figure 6B,C), which was significantly attenuated by KM1608 (600 mg/kg) (Figure 6B). As shown in Figure 6D, the colon weight/length ratio was significantly elevated in the DSS-induced group because of submucosal edema in the inflamed colon. The KM1608 200 and 600 mg/kg groups showed significantly decreased colon weight/length ratios (Figure 6D). 5-ASA (an aminosalicylate anti-inflammatory drug) and prednisolone (a steroid drug) were used as reference drugs. However, the 5-ASA- and prednisolone-treated groups did not show any significant improvement of colitis in this study. Furthermore, we measured MPO activity and pro-inflammatory cytokine levels (IL-6 and TNF-α) to determine the degree of inflammation in the colitis-induced colon. MPO is a peroxidase enzyme mainly found in the neutrophils and is used as an index of neutrophil infiltration. As shown in Figure 7A, MPO activity was decreased in all drug-treated groups, with the DSS-treated group showing a significantly higher MPO activity than that in the normal group. Particularly, MPO activity in the prednisolone- and KM1608 (400 and 600 mg/kg)-treated groups were significantly lower than that in the DSS group (Figure 7A). On the other hand, pro-inflammatory cytokines such as TNF-α and IL-6 levels were elevated in the inflamed tissues, causing colon tissue damage and exacerbating colitis. As shown in Figure 7B,C, TNF-α and IL-6 levels in the colon tissue were higher in the DSS group than in the normal group. In contrast, the levels of these inflammatory cytokines were lower in the KM1608 group than in the DSS group and the reference groups (5-ASA- and prednisolone-treated groups). In addition, KM1608 at 400 and 600 mg/kg inhibited the production of TNF-α and IL-6 more than the reference drug. Based on these results, we speculate KM1608 could be a candidate for a novel therapeutic agent against colitis.

## 3. Materials and Methods

### 3.1. Plant Materials and KM1608 Extraction

Three kinds of herbs, *A. lappa* (China), *T. chebula* (India), and *Z. officinale* (Korea), were purchased from Songrim Muyak (Seoul, Korea) and mixed at a ratio of 2:2:1 (*w*/*w*/*w*). Next, the mixture was subjected twice to reflux extraction using 50% ethanol (80 °C, 3 h). After filtration and solvent removal using vacuum evaporation, the KM1608 was collected (yield: 35–45%). Afterward, it was dissolved in 50% ethanol and filtered through a 0.22 μm membrane filter to obtain a 5 mg/mL KM1608 solution for analysis.

### 3.2. HPLC-UV/DAD Conditions

Quantitative analysis of KM1608 was performed using a Waters UPLC system (Milford, MA, USA) and Waters Acquity UPLC HSS T3 column (100 mm × 2.1 mm, 1.8 μm) (Milford, MA, USA). The mobile phase consisted of water containing 0.1% phosphoric acid (A) and acetonitrile (B). The gradient elution was as follows: 0–1.5 min of 5% (B), 1.5–2 min of 5–12% (B), 2–9 min of 12–13% (B), 9–9.5 min of 13–22% (B), 9.5–11 min of 22–25% (B), and 11–15 min of 25–80% (B). Following gradient elution, the column was washed with 100% (B) for 7 min. The post-running time was 10 min after restoration of the initial condition. The mobile phase flow rate was 0.6 mL/min and the injection volume was 2 μL. A PDA eλ detector was set to an absorbance of 220 nm for dehydrocostus lactone, 254 nm for ellagic acid, and 280 nm for gallc acid, corilagin, 1,2,3,4,6-pentagalloyl glucose, and 6-gingerol. The peaks for dehydrocostus lactone, ellagic acid, gallic acid, corilagin, 1,2,3,4,6-pentagalloyl glucose, and 6-gingerol in KM1608 were compared with their respective standard compounds.

### 3.3. Antibodies and Reagents

Antibodies against, p65 (C-20), p38 (C-20), ERK1 (C-16), JNK (FL), and β-actin (I-19) were purchased from Santa Cruz Biotechnologies (Santa Cruz, CA, USA). Antibodies against phospho-p65 (Ser-536), phospho-ERK1/2 (Thr202/Tyr204), phospho-JNK (Thr-183/Tyr-185), phospho-p38 (Thr-180/Tyr-182), phospho-c-Jun (Ser-73), phospho-Akt (Thr-308), c-Jun (60A8), inducible NO synthase (iNOS, D6B6S) and Akt were purchased from Cell Signaling Technology (Denver, MA, USA). More details of antibody information; Table S1. Mouse IL-6 and TNF-α ELISA kits were purchased from BD Biosciences (Franklin Lakes, NY, USA). Mouse prostaglandin E2 (PGE2), MCP-1, and myeloperoxidase (MPO) ELISA kits were obtained from Abcam (Cambridge, MA, USA), Thermo Fisher Scientific (Waltham, MA, USA), and Hycult Biotechnology (Plymouth Meeting, PA, USA), respectively. LPS from *E. coli* 0111:B4 (ultrapure) was obtained from Invitrogen (San Diego, CA, USA). Dulbecco’s medium (DMEM) was purchased from HyClone (GE Healthcare Life Sciences, Chicago, IL, USA). Fetal bovine serum (FBS) was purchased from ATCC (Manassas, VA, USA). DSS s(colitis grade, molecular weight 36,000–50,000 kDa) was obtained from MP Biomedicals (Santa Ana, CA, USA). 5-aminosalicylic acid (5-ASA), prednisolone, and DMSO, as well as standard compounds of DCL, EA, 6G, gallic acid, corilagin and 1,2,3,4,6-pentagalloyl glucose were purchased from Sigma Aldrich (St. Louis, MO, USA). Solvents for HPLC, including acetonitrile (J. T. Baker™, Phillipsburg, NJ, USA), water (HPLC-grade), and phosphoric acid (85%, HPLC-grade) were purchased from Fisher Scientific (Pittsburgh, PV, USA). All other chemical reagents were purchased from Sigma (St. Louis, MO, USA).

### 3.4. Animal and Cell Culture

Female C57BL/6 mice were purchased from Daehan Bio Link (Seoul, Korea) at 7 weeks of age and acclimatized for 7 days in a specific pathogen-free (SPF) environment under constant conditions (temperature: 23 ± 2 °C; humidity: 50 ± 5%; light/dark cycle: 12 h) at a facility in Kolmar Korea Co., Ltd (Sejong, Korea). All animal studies were performed according to the instructions of the Ethics Committee for Use of Experimental Animals at Kolmar Korea Co., Ltd. (confirmation number: 16-NP-IBD-011-P). The macrophage cell line RAW 264.7 was purchased from the Korean Cell Line Bank (KCLB) (Seoul, Korea) and seeded in DMEM containing 10% heat-inactivated FBS and 1% penicillin-streptomycin obtained from Life Technologies (Waltham, MA, USA). The cells were maintained at 37 °C in a humidified atmosphere with 5% CO_2_.

### 3.5. Induction of Colitis by DSS

Acute colitis was induced in mice for 7 days by adding 1.7% (*w*/*v*) DSS to the drinking water. The mice were measured daily for body weight, stool consistency and rectal bleeding. The normal group received only water without DSS. The DSS group received only drinking water containing 1.7% of DSS. The KM1608 groups received DSS-containing drinking water and KM1608 (200, 400, or 600 mg/kg). The 5-ASA group received DSS-containing drinking water and 5-ASA (200 mg/kg). The prednisolone group received DSS-containing drinking water and prednisolone (5 mg/kg). Carboxymethylcellulose (CMC) solution (0.5%) was used to dissolve KM1608, 5-ASA and prednisolone for in vivo experiments. All drugs were orally administered once daily during the experiment. Animals were sacrificed after 7 days of DSS treatment.

### 3.6. Disease Activity Index (DAI)

Intestinal disease activity was assessed based on body weight loss, diarrhea accompanied by blood and mucus, and colonic shortening. DAIs were assessed using a scoring system (Table 2) as described by Murthy et al. [28] with little modification, and were calculated using the following formula: DAI = (weight loss score) + (stool consistency score) + (rectal bleeding score).

### 3.7. Cell Viability

RAW 264.7 macrophages were treated with the indicated concentration of KM1608 for 2 h. The cells were subsequently treated with LPS (250 ng/mL) for 18 h. Following treatment, 20 μL of CCK-8 solution was added to the cells and the cells were further incubated at 37 °C, 5% CO_2_ for the evaluation of cytotoxic effects. KM1608 (herbal formulation) was dissolved in dimethyl sulfoxide (DMSO) prior to use for in vitro experiments, and the final concentration was kept at <0.1%.

### 3.8. Determination of Nitrite, IL-6, MCP-1, TNF-a, and MPO Production

RAW 264.7 cells (1 × 10^5^ cells/well, 96-well plate) were treated with KM1608 at various concentrations for 2 h and were subsequently treated with LPS (250 ng/mL). After 18 h of incubation, nitrite production was estimated by using Griess reagent and a standard curve previously prepared using sodium nitrite purchased from Promega (Promega, Fitchburg, WI, USA). The cell supernatants (50 μL) were mixed with the same volume of Griess reagent and the absorbance was measured at 520 nm using a microplate reader (Molecular Devices Co., San Jose, CA, USA). IL-6 and MCP-1 levels in the cell supernatants were evaluated using a sandwich enzyme-linked immunosorbent assay (ELISA) kit according to the manufacturer’s instruction. For the analysis of IL-6, TNF-α, and MPO in colitis-induced colon tissue, colon tissue samples were suspended in lysis buffer (Intron, Seoul, Korea) and ground using a homogenizer (Scilogex, Rocky hill, CT, USA). The supernatant was collected by centrifugation (10,000 rpm, 20 min, 4 °C). The IL-6, TNF-α, and MPO levels in the supernatant were measured using ELISA kits according to the manufacturer’s instructions.

### 3.9. Cell Lysate Preparation and Immunoblotting

RAW 264.7 cells were treated with the indicated concentrations of KM1608 for 2 h and subsequently treated with LPS for 1 h or 18 h. Following treatment, the cells were washed with cold PBS and lysed with cold RIPA buffer (50 mM Tris-HCl pH 7.4, 150 mM NaCl, 0.25% deoxycholate, 1% NP-40, and 1 mM EDTA) containing 1 mM DTT, 1 mM PMSF, 1 mM sodium orthovanadate, and 10 mM β-glycerophosphate. Cell lysis, supernatant collection, protein quantification, protein electrophoresis, protein transfer, and membrane development were all performed as described previously [29]. The relative protein expression compared to controls was quantified using the Image J (National Institutes of Health, Bethesda, MD, USA).

### 3.10. Real-Time Reverse Transcription PCR (qRT-PCR)

Total cellular RNA was isolated by using the RNeasy Mini Kit (Qiagen, Germantown, MD, USA) [30]. RNA was converted into cDNA by using the RevertAid First Strand cDNA Synthesis kit (Fermentas, Waltham, MA, USA). PCR was performed using the Power SYBR Green PCR Master Mix or PowerUp SYBR PCR Master Mix (Applied Biosystems, Waltham, CA, USA) with sense and antisense primers. The primers for iNOS were 5′-ACATCGACCCGTCCACAGTAT-3′ and 5′-CAGAGGGGTAGGCTTGTCTC-3′ (NM_001313921.1); for IL-6 were 5′-GAGGATACCACTCCCAACAGACC-3′ and 5′-AAGTGCATCATCGTTGTTCATACA-3′ (NM_031162.2); for TNF-α were 5′-CTGTAGCCCACGTCGTAGC-3′ and 5′-TTGAGATCCATGCGTTG-3′ (NM_013693.3); for MCP-1 were 5′-GTCCCTGTCATGCTTCTGG-3′ and 5′-GCGTTAACTGCATCTGGCT-3′ (NM_011333.3); and β-actin was used as a housekeeping gene, with primers 5′-GGCATTGTTACCAACTGGGACGAC-3′ and 5′-CCAGAGGCATACAGGGACAGCACAG-3′ (NM_007393.5). The amplification conditions were 95 °C for 10 min, followed by 40 cycles of 95 °C for 15 s and 60 °C for 1 min (IL-6) or 95 °C for 15 s, 58 °C for 20 s and 72 °C for 1 min (iNOS, TNF-α, and β-actin) using 7500 real time PCR system (Applied Biosystems) or 95 °C for 20 s and 95 °C for 1 s, 58 °C for 30 s (MCP-1) using Quant Studio 3 real time PCR system (Applied Biosystems). Melt curve of each primers; See Figure S2.

### 3.11. Statistical Analysis

The data are expressed as the mean ± standard deviation of duplicate or triplicate experiments. Statistical analysis of the in vivo experimental data was performed using one-way ANOVA and the Tukey post-hoc test, with statistical significances at * *p* < 0.05 and ** *p* < 0.01. For the in vitro experiment data, statistical analysis was performed using the Student’s *t*-test with ^#^ *p* < 0.05 as the statistical significance.

## 4. Conclusions

In this study, to investigate the anti-inflammatory effect of the novel herbal formulation KM1608, we examined the expression levels of NO, iNOS, IL-6, MCP-1, and TNF-α in LPS-stimulated RAW264.7 macrophages. We confirmed that KM1608 reduced the LPS-induced increase in NO level and that KM1608 also inhibited iNOS gene and protein expression in a concentration-dependent manner. In addition, IL-6 TNF-α and MCP-1 production during the inflammation process were also inhibited by KM1608. In DSS-induced colitis model, as indicated by the DAI, colon length, and colon weight, KM1608 alleviated colitis more than the 5-ASA and prednisolone, the reference drugs for colitis. Moreover, KM1608 also suppressed the expression of inflammatory cytokines (TNF-α, IL-6) and MPO in the DSS-induced colon tissues. Taken together, the novel herbal formulation KM1608 exerted ameliorative effects on ulcerative colitis through anti-inflammatory responses. Therefore, future studies need to consider not only single natural-products but also herbal formulations for the development of anti-inflammatory drugs.

## Figures and Tables

**Table 1:** Contents of representative compounds in KM1608.

Plant	Active Compound	Content (%)
*Aucklandia lappa* DC.	dehydrocostus lactone	0.68–1.2
*Terminalia chebula* Retz	ellagic acid	0.94–1.5
*Zingiber officinale* Roscoe	6-gingerol	0.1–0.2

**Table 2:** Disease Activity Index (DAI) scoring system.

Score	Weight Loss	Stool Consistency	Occult/Gross Rectal Bleeding
0	None	Normal	Normal
1	1–5%	-	-
2	5–10%	Loose stool	Hemoccult
3	10–20%	-	-
4	>20%	Diarrhea	Gross bleeding

## Supplementary Materials

The following are available online, Figure S1: Whole western blot membranes of Figure 5C, Figure S2: Melt curve of Figure 3, Table S1: Antibody source and usage information for western blots.

## References

[1] Bingham C.O. (2002). The pathogenesis of rheumatoid arthritis: Pivotal cytokines involved in bone degradation and inflammation. J. Rheumatol. Suppl..

[2] Medzhitov R. (2008). Origin and physiological roles of inflammation. Nature.

[3] Lundberg I.E. (2000). The role of cytokines, chemokines, and adhesion molecules in the pathogenesis of idiopathic inflammatory myopathies. Curr. Rheumatol. Rep..

[4] Payne D.N.R. (2003). Nitric oxide in allergic airway inflammation. Curr. Opin. Allergy Clin. Immunol..

[5] Baumgart D.C., Carding S.R. (2007). Inflammatory bowel disease: Cause and immunobiology. Lancet.

[6] Kornbluth A., Sachar D.B. (2004). Ulcerative colitis practice guidelines in adults (update): American college of gastroenterology, practice parameters committee. Am. J. Gastroenterol..

[7] Choi C.H., Kim Y.H., Kim Y.S., Ye B.D., Lee K.M., Lee B.I., Jung S.A., Kim W.H., Lee H. (2012). IBD Study Group of the Korean Association for the Study of Intestinal Diseases. Guidelines for the management of ulcerative colitis. Korean J. Gastroenterol..

[8] Jeen Y.T., Kim J.H. (2009). Advances in ulcerative colitis therapy. Korean J. Med..

[9] Kim S.Y., Park J.W., Ryu B.H. (2013). Effects of Auklandia Lappa on dextran sulfate sodium-induced murine colitis. Korean J. Orient. Int. Med..

[10] Kim D.S., Kim S.H., Kee J.Y., Han Y.H., Park J., Mun J.G., Joo M.-J., Jeon Y.-D., Park S.-H., Park S.-J. (2017). Eclipta prostrata improves dss-induced colitis through regulation of inflammatory response in intestinal epithelial cells. Am. J. Chin. Med..

[11] Yoon W.H., Lee K.H. (2017). Anti-inflammatory, anti-arthritic and analgesic effect of the herbal extract made from bacopa monnieriis, cassia fistula and phyllanthus polyphyllus. Nat. Prod. Sci..

[12] Shin M.S., Park J.Y., Lee J., Yoo H.H., Hahm D.H., Lee S.C., Lee S., Hwang G.S., Jung K., Kang K.S. (2017). Anti-inflammatory effects and corresponding mechanisms of cirsimaritin extracted from *Cirsium japonicum* var. *maackii* Maxim. Bioorg. Med. Chem. Lett..

[13] Lee K.H., Yoon W.H. (2011). Preventive effects of co-treatment with fucoidan and lutein on the development of Inflammatory Bowel Disease in DSS mouse model. Nat. Prod. Sci..

[14] Jang S.E., Choi J.R., Han M.J., Kim D.H. (2016). The preventive and curative effect of cyanidin-3β-d-glycoside and its metabolite protocatechuic acid against TNBS-induced colitis in mice. Nat. Prod. Sci..

[15] Kang C.S., Tae J., Ham S.H., Kim D.K., Lee Y.M., Lee K.S., Yun Y.G. (2007). Administration of aqueous extract of *Schizandra chinensis* fruit inhibits the experimental colitis in mice. Nat. Prod. Sci..

[16] Singh P., Malhotra H. (2017). *Terminalia chebula*: A review pharmacognistic and phytochemical studies. Int. J. Recent Sci. Res..

[17] Li A., Sun A., Liu R. (2005). Preparative isolation and purification of costunolide and dehydrocostuslactone from *Aucklandia lappa* Decne by high-speed counter-current chromatography. J. Chromatogr. A.

[18] Ali B.H., Blunden G., Tanira M.O., Nemmar A. (2008). Some phytochemical, pharmacological and toxicological properties of ginger (*Zingiber officinale* Roscoe): A review of recent research. Food Chem. Toxicol..

[19] Lee H.J., Kim N.Y., Jang M.K., Son H.J., Kim K.M., Sohn D.H., Lee S.H., Ryu J.H. (1999). A sesquiterpene, dehydrocostus lactone, inhibits the expression of inducible nitric oxide synthase and TNF-α in LPS-activated macrophages. Planta Med..

[20] Pandey M.M., Rastogi S., Rawat A.K.S. (2007). Saussurea costus: Botanical, chemical and pharmacological review of an ayurvedic medicinal plant. J. Ethnopharmacol..

[21] Maged R., Nordin N., Abdulla M.S. (2013). Anti-inflammatory effects of zingiber officinale roscoe involve suppression of nitric oxide and prostaglandin E2 production. Zanco J. Med. Sci..

[22] Mansouri M.T., Hemmati A.A., Naghizadeh B., Mard S.A., Rezaie A., Ghorbanzadeh B. (2015). A study of the mechanisms underlying the anti-inflammatory effect of ellagic acid in carrageenan-induced paw edema in rats. Indian J. Pharmacol..

[23] Pandurangan A.K., Mohebali N., Norhaizan M.E., Looi C.Y. (2015). Gallic acid attenuates dextran sulfate sodium-induced experimental colitis in BALB/c mice. Drug Des. Devel. Ther..

[24] Xiao H.T., Lin C.Y., Ho D.H., Peng J., Chen Y., Tsang S.W., Bian Z.X. (2013). Inhibitory effect of the gallotannin corilagin on dextran sulfate sodium-induced murine ulcerative colitis. J. Nat. Prod..

[25] Zhang J., Li L., Kim S.H., Hagerman A.E., Lü J. (2009). Anti-cancer, anti-diabetic and other pharmacologic and biological activities of penta-galloyl-glucose. J. Pharm. Res. Int. Pharm. Res..

[26] Hossen M.J., Hong Y.D., Baek K.S., Yoo S., Hong Y.H., Kim J.H., Lee J.O., Kim D., Park J., Cho J.Y. (2017). In vitro antioxidative and anti-inflammatory effects of the compound K-rich fraction BIOGF1K, prepared from Panax ginseng. J. Ginseng Res..

[27] Hommes D.W., Peppelenbosch M.P., Van Deventer S.J.H. (2003). Mitogen activated protein (MAP) kinase signal transduction pathways and novel anti-inflammatory targets. Gut.

[28] Murthy S.N.S., Cooper H.S., Shim H., Shah R.S., Ibrahim S.A., Sedergran D.J. (1993). Treatment of dextran sulfate sodium-induced murine colitis by intracolonic cyclosporin. Digest. Dis. Sci..

[29] Shin M.S., Song J.H., Choi P., Lee J.H., Kim S.Y., Shin K.S., Kang K.S. (2018). Stimulation of innate immune function by panax ginseng after heat processing. J. Agric. Food Chem..

[30] Lee H., Kim J., Park J.Y., Kang K.S., Park J.H., Hwang G.S. (2017). Processed Panax ginseng, sun ginseng, inhibits the differentiation and proliferation of 3T3-L1 preadipocytes and fat accumulation in Caenorhabditis elegans. J. Ginseng Res..

