# Supplementary material for: Beneficial Effect of Herbal Formulation KM1608 on Inflammatory Bowl Diseases: A Preliminary Experimental Study
Source: Molecules. 2018 Aug 17;23(8):2068. doi: 10.3390/molecules23082068 (PMC6222370; doi:10.3390/molecules23082068)
Supplement: Supplementary file 1 [file molecules-23-02068-s001.pdf]

Supplemental experimental Information

1. **Whole western blot membranes of Fig 5C.**

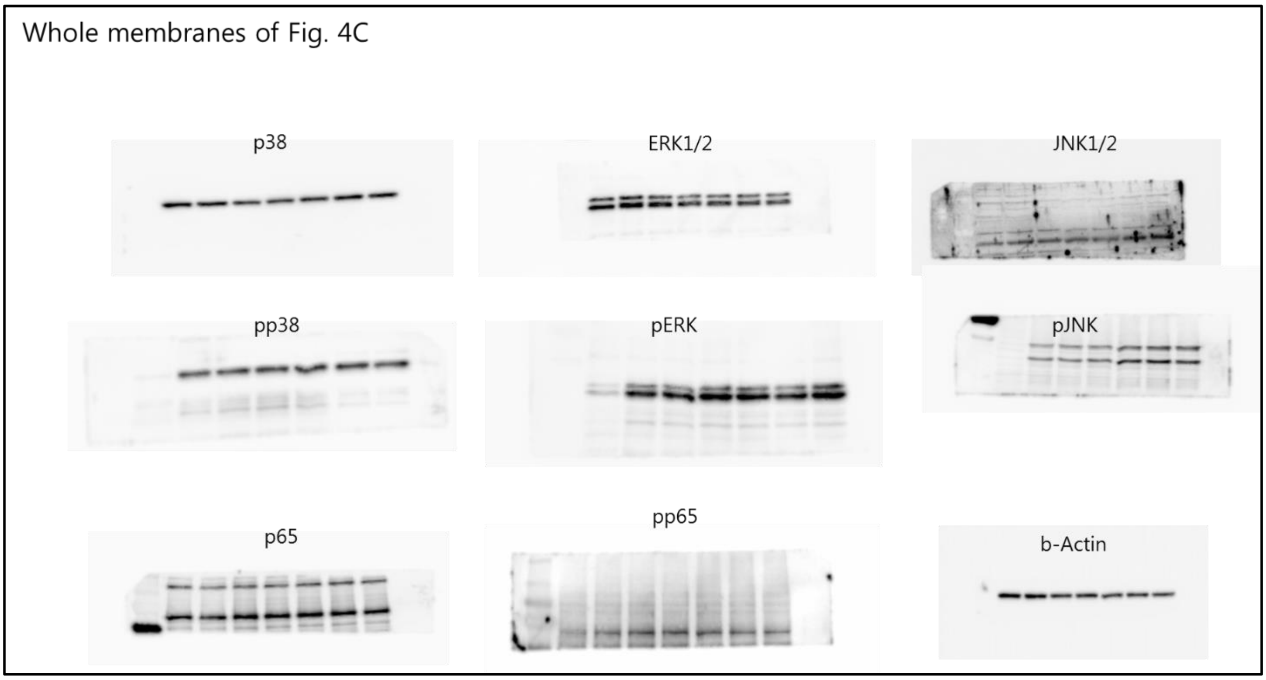

2. **Melt curve of Fig 3**

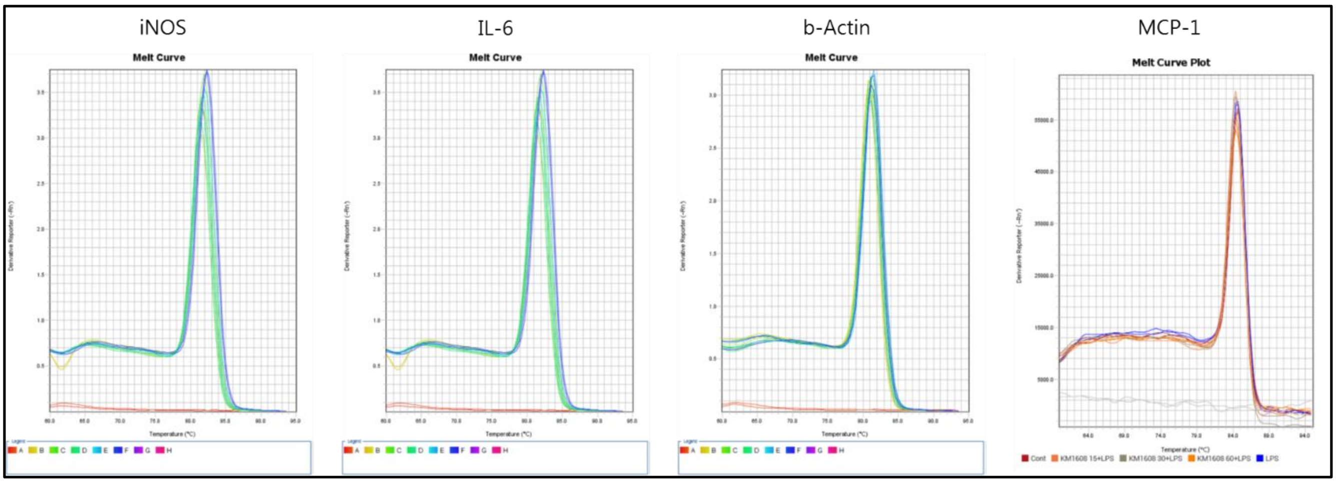

3. Antibody source and usage information for western blots.

Antibody	Dilution	Catalog number	Sources
iNOS (D6B6S)	1:1000	#13120	Cell Signaling Technology, Denver, MA, USA
Phospho-ERK1/2 (T202/Y204)	1:2500	#4370	Cell Signaling Technology, Denver, MA, USA
Phospho-JNK (T183/Y185)	1:2500	#4671	Cell Signaling Technology, Denver, MA, USA
Phospho-p38 (T180/Y182)	1:2500	#4511	Cell Signaling Technology, Denver, MA, USA
Phospho-p65 (S536)	1:2500	#3033	Cell Signaling Technology, Denver, MA, USA
Phospho-c-Jun (S73)	1:2500	#13038	Cell Signaling Technology, Denver, MA, USA
phospho-Akt (T308)	1:2000	#13038	Cell Signaling Technology, Denver, MA, USA
c-Jun (60A8)	1:2000	#9165	Cell Signaling Technology, Denver, MA, USA
Akt	1:2000	#9272	Cell Signaling Technology, Denver, MA, USA
ERK1 (C-16)	1:3000	SC-93	Santa Cruz Biotechnologies, Santa Cruz, CA, USZ
JNK (FL)	1:1000	SC-571	Santa Cruz Biotechnologies, Santa Cruz, CA, USZ
p38 (C-20)	1:2500	SC-535	Santa Cruz Biotechnologies, Santa Cruz, CA, USZ
p65 (C-20)	1:2500	SC-372	Santa Cruz Biotechnologies, Santa Cruz, CA, USZ
b-Actin (I-19)	1:3000	SC-1616	Santa Cruz Biotechnologies, Santa Cruz, CA, USZ
